# The origin of myocardial passive stiffness: more than the sum of its parts?

**DOI:** 10.1007/s00424-024-02936-x

**Published:** 2024-02-29

**Authors:** Martina Krüger

**Affiliations:** 1 Institute of Cardiovascular Physiology, Medical Faculty and University Hospital Düsseldorf, Heinrich Heine University Düsseldorf, Universitätsstr. 1, 40225 Düsseldorf, Germany; 2 CARID, Cardiovascular Research Institute Düsseldorf, University Hospital Düsseldorf, Heinrich Heine University Düsseldorf, Düsseldorf, Germany

This comment summarizes and discusses the key findings of the recent publication by Loescher, C.M., Freundt, J.K., Unger, A. et al. Titin governs myocardial passive stiffness with major support from microtubules and actin and the extracellular matrix. Nat Cardiovasc Res 2, 991–1002 (2023). 10.1038/s44161-023-00348-1. It introduces the background of the addressed issue, highlights the strength of the study and discusses remaining questions.

Comment:

The stiffness of the myocardium plays a crucial role in determining cardiac function, for which passive diastolic filling of the ventricles is essential. It is also important for systolic function, due to the load-dependent activation through the Frank-Starling mechanism. Myocardial passive stiffness is largely determined by the extracellular matrix and the sarcomere filament titin, and changes in expression and posttranslational modification of both are important for pathologically impaired diastolic function [6, 10]. More recent studies demonstrated that the intracellular network of intermediate filaments and microtubules, which is involved in mechanical signaling, intracellular transport, and structural organization of myofibrils, also contributes to cardiomyocyte passive stiffness in healthy and diseased hearts [2]. This has once again fueled ongoing debates about which of the extracellular and intracellular components involved plays the key role [1]. At first glance, this debate may appear to be of purely academic interest, but it may be of considerable clinical importance, as it could set the focus for further studies and therapeutic approaches to heart failure.

Loescher et al. have revisited the topic with a very elegant and comprehensive methodological approach [7]. By using a genetic mouse model for titin cleavage [9], the authors were able to specifically disrupt the titin filament and investigate its involvement in the passive stiffness of ventricular fiber bundles and single cardiomyocytes under different load conditions. Step by step, the authors specifically removed extracellular matrix, microtubules and actin using different protocols and analyzed their relative contribution to passive stiffness. Importantly, unlike many others before, Loescher et al. went one step further and broke down the viscoelastic properties of the myocardium into two defined sub-aspects: the velocity-insensitive = elastic component and the velocity-sensitive = viscous component. The results of the study are truly exciting and emphasize that all together titin, microtubules, actin and the extracellular matrix significantly influence passive stiffness. Moreover, the study provides a detailed breakdown of the players and their significance for different aspects of passive forces. It turns out that microtubules have a stronger effect on viscous forces, whereas titin significantly contributes under all conditions and dominates the elastic forces at both low and high strains. This confirms that titin is a major determinant of the passive stiffness relevant for diastolic filling and that targeted manipulation of titin is a promising concept to counteract pathological changes in both the viscous and elastic components of passive stiffness.

Although the study revealed the contribution of different players under controlled physiological conditions, it did not resolve but rather stimulated the debate on the relative contributions during heart failure. In the intact myocardium, all protein networks and filament systems interact in a highly complex manner. Therefore, manipulating a single component will most likely affect all others. It should further be considered that the passive stiffness is dynamically modulated during disease progression, e.g. by posttranslational modification [6, 3]. In mouse hearts it was shown that titin-mediated passive tension is significantly increased within hours after ischemia/reperfusion, whereas changes in collagen expression were observed only a few days after the ischemic event [5, 4]. In contrast to these early adaptations, hearts from patients with end-stage ischemic heart failure showed increased collagen expression and microtubule-dependent changes to cardiomyocyte passive stiffness, whereas titin-based passive tension was reduced due to altered titin isoform composition [3, 8]. These observations add a temporal component to the already complex relationship of all contributors, which must be considered when developing approaches to improve myocardial passive stiffness. Taken together, it once again holds true what Aristotle said: the whole seems to be more than the sum of its parts.

## Data Availability

No datasets were generated or analysed during the current study.
